# The First Case of a Cat Infected with *Burkholderia pseudomultivorans*, a Member of the *Burkholderia cepacia* Complex

**DOI:** 10.3390/vetsci11110559

**Published:** 2024-11-12

**Authors:** Yuji Fujii, Akihisa Suwa, Yuzo Tsuyuki, Kumiko Koyama, Junko Nio-Kobayashi, Kentaro Yoshii

**Affiliations:** 1 National Research Center for the Control and Prevention of Infectious Diseases, Nagasaki University, 1-12-4 Sakamoto, Nagasaki 852-8523, Japan; koyamakumiko@nagasaki-u.ac.jp (K.K.); niojun@nagasaki-u.ac.jp (J.N.-K.); 2 Suwa Animal Hospital, 2-149-3 Kokura, Fukuoka 816-0824, Japan; as0718y@gmail.com; 3 Division of Clinical Laboratory, Sanritsu Zelkova Veterinary Laboratory, 3-5-5 Ogibashi, Tokyo 135-0011, Japan; y-tsuyuki@san-g.com

**Keywords:** *Burkholderia pseudomultivorans*, cat, genetic analysis, *recA*, sepsis

## Abstract

The *Burkholderia cepacia* complex (Bcc) is a group of bacteria with similar biological properties which are an important cause of opportunistic infections. Identification of the Bcc species is important to understand clinical outcomes in human patients. However, there are few studies of Bcc species infecting companion animals such as cats. This report shows for the first time an infection with *B. pseudomultivorans*, a member of Bcc, in veterinary medicine. A 9-year-old, FIV-positive, mixed-breed cat was subjected to immunosuppressive therapy, developed septic shock, and died. In the bone marrow specimen collected postmortem, numerous short bacilli—some of which were phagocytosed by leukocytes—were observed. By bacteriological examination, the Bcc-like bacteria were isolated from the bone marrow sample. Genetic analysis showed that the *recA* gene sequence detected from the present case exhibited the highest homology (98–100%) to that of *B. pseudomultivorans*. Based on these examinations, the present case was diagnosed as *B. pseudomultivorans* infection. The findings of this study highlight the need for investigation of the Bcc species in veterinary practice to help in treatment decisions and the prognostic evaluation of Bcc infections.

## Simple Summary

The *Burkholderia cepacia* complex (Bcc) is a group of more than 20 closely related species associated with opportunistic infections. In this report, we describe a species identification of a Bcc strain detected from a cat with sepsis. A 9-year-old, FIV-positive, mixed-breed cat died during the immunosuppressive therapy. In the bone marrow specimen collected postmortem, numerous short bacilli were observed. Bacteriological examination and genetic analysis revealed that the bacterial species isolated from the cat was *B. pseudomultivorans*, a member of Bcc. This is the first report of infection with *B. pseudomultivorans* in companion animals. The findings of this study indicate that further investigations of Bcc infections should be performed in veterinary medicine.

## 1. Introduction

The *Burkholderia cepacia* complex (Bcc), previously classified as a part of the *Pseudomonas* genus, is a group of Gram-negative bacteria comprising more than 20 species, including *B. cepacia*, *B. multivorans*, *B. cenocepacia*, and *B. pseudomultivorans* [1,2]. In human medicine, Bcc was first isolated from patients with cystic fibrosis in 1977 [3]. Subsequent investigations revealed that Bcc causes pneumonia and bloodstream infections, mainly in immunocompromised patients [4]. In veterinary medicine, Bcc causes deep-seated pyoderma and occasionally sepsis in immunosuppressed dogs and cats [5,6,7]. Notably, Bcc dwells in a wide variety of ecological niches and possesses natural resistance mechanisms to most of the available antibiotics [1]. Such properties make the eradication of Bcc in medical settings very difficult. Indeed, outbreaks and nosocomial transmission of Bcc are often reported [4,8]. Bcc is, therefore, recognized as one of the pathogens causing opportunistic infections.

The severity risk in Bcc infections varies widely across species, and therefore, the identification of the Bcc species is important for clinical treatment, prognostic evaluation, and epidemiologic studies [2,9]. Because Bcc bacteria are difficult to phenotypically classify owing to their similar biological characteristics, molecular biology methods—especially analysis of housekeeping genes such as the *recA* gene—are useful for the identification of the Bcc species [2,10]. With the development of genome-based species identification methods, new Bcc species, including *B. psuedomultivorans*, that are pathogenic in humans have been successively revealed over the past decades [11]. On the other hand, Bcc infections in companion animals have largely not been examined. Therefore, which Bcc species cause disease in dogs and cats remains unknown. In this study, we genetically analyzed a Bcc strain detected in a cat with sepsis, demonstrating *B. psuedomultivorans* infection in veterinary medicine for the first time.

## 2. Case Description

**Figure 1:**
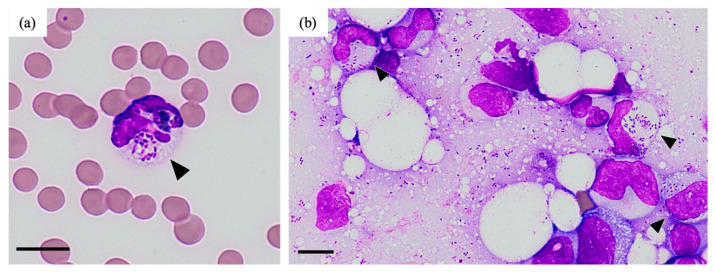
Postmortem cytology (day 41). Short bacilli, some of which were phagocytosed by leukocytes (indicated by arrowheads), were observed in peripheral blood (**a**) and bone marrow (**b**). Scale bar indicates 10 µm.

A 9-year-old male neutered mixed-breed cat showed decreased activity and anorexia for one month. The body weight was 4.9 kg (6–7 kg a year earlier), and the temperature was 37.9 °C. The cat was positive for feline immunodeficiency virus (FIV) antibodies (date of test: unknown). This case was kept indoors with other cats. At the time of the visit, the case was already treated with prednisolone because anemia was found at another hospital (detailed information was not obtained). Blood examination at the initial visit (day 1) revealed non-regenerative anemia (hematocrit 18.0%, reticulocyte counts 4.0 × 10^4^/μL) and thrombocytopenia (platelet counts 5.2 × 10^4^/μL) (Table 1). There were no significant findings in peripheral blood smears on the same day. Bone marrow examination on day 1 revealed no abnormalities in the morphology or maturation of each blood cell lineage. In addition, there were no findings suggestive of bacterial infection. Based on these examinations, we tentatively diagnosed the cat with immune-mediated cytopenia, such as immune-mediated hemolytic anemia (IMHA) and precursor-targeted immune-mediated anemia (PIMA), and administered immunosuppressive therapy with prednisolone and cyclosporine (3 and 10 mg/kg/day, respectively) from day 1. In addition, because three *Mycoplasma* spp. (*M. haemofelis*, ‘*Candidatus M. haemominutum*’, and ‘*Candidatus M. turicensis*’) genes were detected by real-time PCR (performed at IDEXX Laboratories, inc.) in the day 1 blood sample, we treated the case with doxycycline (10 mg/kg/day) from day 8. Since the hematocrit levels were elevated (day 8: 27.8%, day 22: 29.0%), the dose of prednisolone was gradually reduced. However, the progression of pancytopenia and worsening of the general condition (failure to stand, loss of appetite, and tachypnea) were suddenly observed on day 41 (treatment: prednisolone [1 mg/kg/day], cyclosporine [10 mg/kg/day], and doxycycline [10 mg/kg/day]). More specifically, a neutrophil left shift with toxic granule (band neutrophils 0.8 × 10^3^/µL, segmented neutrophils 1.1 × 10^3^/µL) and multiple organ failure (alanine transaminase [ALT] 149 U/L, blood urea nitrogen [BUN] 78.8 mg/dL, total bilirubin [TBil] 3.6 mg/dL, creatinine [Cre] 2.6 mg/dL, glucose [GLU] 37 mg/dL) were observed (Table 1), and the patient died on the same day. The cytology examination of the blood and bone marrow specimens (sampled before and after death, respectively) revealed the presence of short bacilli, some of which were phagocytosed by leukocytes (Figure 1). Further postmortem examinations were not carried out due to the animal owner’s wishes. From the above findings, we concluded that the cause of death of this cat was septic shock from bacterial infection.

The infected bacteria (strain name: LMN08) were isolated from the postmortem sample of the bone marrow using a standardized method. Analysis of the matrix-assisted laser desorption/ionization–time of flight mass spectrometry (MALDI-TOF MS) showed that this isolate showed characteristics similar to those of the Bcc species. Minimum inhibitory concentrations (MICs, µg/mL) of 16 antimicrobials (minocycline, ceftazidime, levofloxacin, trimethoprim–sulfamethoxazole, meropenem, piperacillin, gentamicin, tobramycin, amikacin, cefozopran, cefepime, ciprofloxacin, fosfomycin, azidothymidine, tazobactam-piperacillin, and imipenem) were determined using the broth microdilution method (NC-NF3J; Beckman Coulter, Tokyo, Japan) recommended in the Clinical and Laboratory Standards Institute (CLSI) guideline for Bcc [12]. Susceptibility quality control was performed using *P. aeruginosa* ATCC 27853. The drug susceptibility of the LMN08 isolate is shown in Table 2.

**Figure 2:**
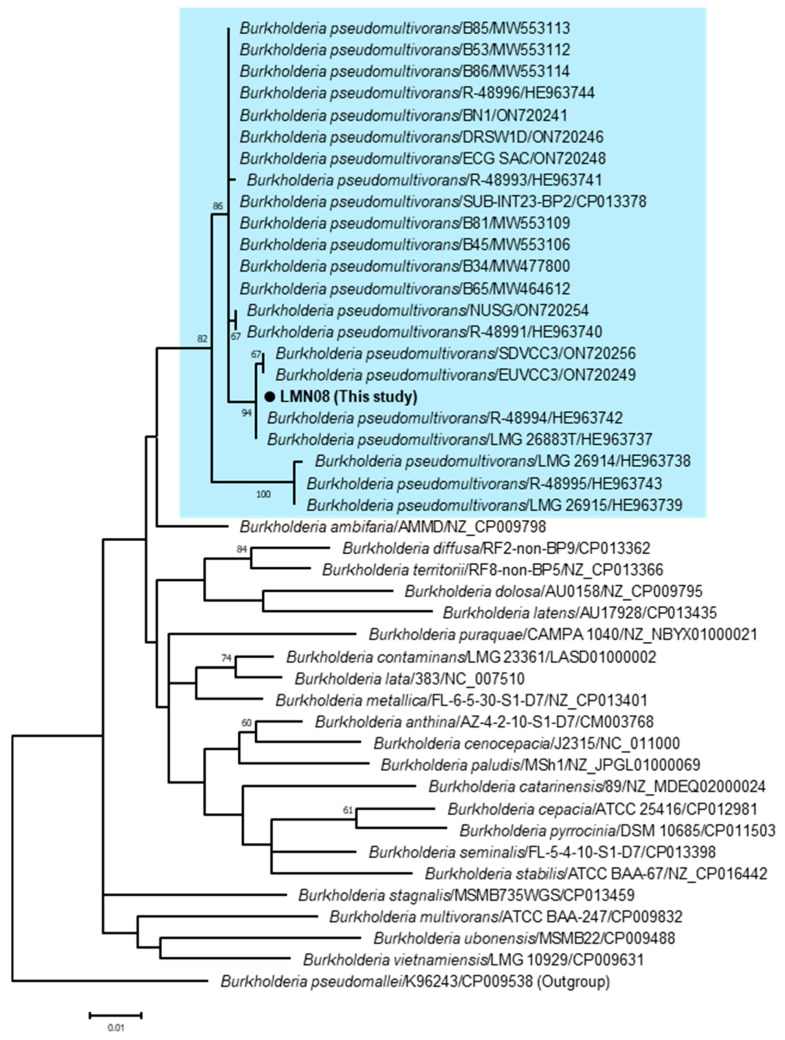
Phylogenetic tree based on partial nucleotide sequences of the *recA* gene (730 bp) of the LMN08 strain and previously reported *Burkholderia* strains. The sequence alignment and construction of the phylogenetic tree were performed using MEGA version 10 software [14]. A phylogenetic tree was reconstructed using the maximum-likelihood method with 1000 bootstrap replicates. The fittest substitution model for the data set was selected based on the Bayesian information criterion score. The model used in this study was the Tamura 3-parameter incorporating a gamma distribution and invariant sites (T92+G+I). The scale bar indicates the number of nucleotide substitutions per site, and bootstrap values (1000 replicates) above 60 are shown. The strain name and GenBank accession number are shown next to the species names. The background of *B. pseudomultivorans* is highlighted in light blue.

To identify the bacterial species of the LMN08 strain, we analyzed the *recA* gene, a housekeeping gene of Bcc. Briefly, DNA was extracted from the postmortem bone marrow sample using the DNeasy Blood and Tissue kit (QIAGEN, Hilden, Germany) following the manufacturer’s instructions. The extracted DNA was amplified by conventional PCR using KOD one (TOYOBO, Osaka, Japan) with the *recA* gene-specific primers BUR1 and BUR2 [13]. The PCR product was separated on 1.2% agarose gels and then purified with the Wizard SV Gel and PCR Clean-Up System (Promega, Madison, WI, USA). The purified PCR product was sequenced by the BigDye Terminator v3.1 Cycle Sequencing kit (Applied Biosystems, Foster City, CA, USA) on the ABI PRISM 3500 DNA analyzer (Applied Biosystems). The above PCR primers were used as sequencing primers. The nucleotide sequence obtained in this study was deposited in GenBank under the accession number LC834785. A similarity analysis using BLASTn “https://blast.ncbi.nlm.nih.gov/Blast.cgi (accessed on 2 October 2024)” revealed that the sequence of the LMN08 strain (730 bp) showed the highest rate (98–100%) with the *recA* gene sequence of *B. pseudomultivorans*. On a phylogenetic tree of the *recA* gene, the LMN08 strain was clustered together with the previously reported strains of *B. pseudomultivorans* (Figure 2). These findings indicated that the Bcc species isolated from the cat was *B. pseudomultivorans*.

## 3. Discussion

The cytological and genetic analyses (Figure 1 and Figure 2) led to the diagnosis of the present case as a *B. pseudomultivorans* infection and subsequent sepsis. *B. pseudomultivorans* is a novel Bcc species first reported in 2013 and occasionally identified in human patients with cystic fibrosis, a disease associated with immunodeficiency and bacteremia [11,15,16]. These facts indicate that *B. pseudomultivorans*, like other Bcc species, causes opportunistic infections in humans. Because this case was an FIV-infected individual and on immunosuppressive therapy, the cat was likely immunocompromised at the time of illness onset. Therefore, it seems reasonable that a state of reduced immunity contributed to the establishment of the *B. pseudomultivorans* infection in this cat, similar to human patients.

The route of infection in this case remains uncertain. Because Bcc is widely distributed in the environment [1], we cannot rule out the possibility that the bacteria invaded through the scar from the bone marrow puncture performed at the initial visit. In addition, the contamination of medical equipment, disinfectants, and pharmaceutical formulations has been directly related to some outbreaks of Bcc infection in humans [4]. In addition, many recent reports have demonstrated that the Bcc bacteria were isolated from disinfectant solutions and unopened ultrasound gels, indicating that these supplies may be a source of Bcc infection in a veterinary setting [5,17]. Given such a wide variety of possible routes of infection, it was difficult to determine how the cat, a solitary case, was infected with *B. psuedomultivorans*. In the future, information on Bcc epidemiology and similar cases will be important in discussing the control of Bcc infections in veterinary medicine.

In this study, a genome-based species identification revealed that *B. pseudomultivorans* was infectious to and pathogenic in a cat. The Bcc species identification has contributed to progress not only in bacterial taxonomy and epidemiology but also in the clinical field. For example, human patients infected with *B. cenocepacia* are at higher risk than those infected with other Bcc species [18]. These facts indicated the possibility that Bcc species identification is beneficial in determining treatment strategy and in assessing prognosis. On the other hand, it remains unclear whether Bcc bacterial species are associated with clinical signs and outcomes in companion animals. In the present case, there were no signs of the cutaneous manifestations that were observed in most of the previously reported cases of Bcc infection in dogs and cats [5,6,7]. To determine whether the clinical features observed in the present case, including laboratory findings and drug susceptibility (Table 1 and Table 2), are representative of *B. psuedomultivorans* infection in companion animals, the relationship between the Bcc species and pathogenesis should be clarified.

The bacterial isolate from this cat was susceptible to minocycline, ceftazidime, levofloxacin, trimethoprim–sulfamethoxazole, and meropenem (Table 2). These results are generally consistent with those of the susceptibility testing of Bcc isolates from cats in the previous study [5]. In addition, this previous study confirmed one case that showed a favorable outcome after the administration of ciprofloxacin, although the susceptibility of this drug is not standardized in the CLSI guideline for Bcc [12]. Given these findings, it might be possible that appropriate therapeutic intervention with one of the agents described above may have improved the outcome in this case. On the other hand, this case was treated with doxycycline until death. It remains uncertain why the use of this drug was not effective in this case, even though the isolate was susceptible to minocycline, which is classified as a tetracycline antibiotic, as well as doxycycline. One possible explanation is that differences in the disposition of these drugs in the body (e.g., drug distribution to the bone marrow, the presumed principal lesion in this case) might be involved. Further studies based on an accumulation of cases are needed to establish treatment strategies for Bcc infections in cats.

## 4. Conclusions

This is the first report of *B. psuedomultivorans* infection in a cat. The findings of this study highlight the need for the identification of Bcc species in veterinary practice to help in treatment decisions and the prognostic evaluation of Bcc infections.

## Figures and Tables

**Table 1:** Blood parameters at days 1 and 41.

Parameter	Reference Interval	Unit	Day 1	Day 41
Complete blood counts				
RBC	6.5–12.2	×10^6^/µL	3.1	2.2
Hct	30.3–52.3	%	18	12.5
HGB	9.8–16.2	g/dL	5.6	4.2
MCV	35.9–53.1	fL	57.5	56.8
MCHC	28.1–35.8	g/dL	31.1	33.6
Reticulocyte	<5	×10^4^/µL	4	0
WBC	2.9–17	×10^3^/µL	5.2	2.3
band-N	<0.3	×10^3^/µL	0	0.8
seg-N	2.3–10.3	×10^3^/µL	4.3	1.1
lym	0.9–6.9	×10^3^/µL	0.5	0.3
mon	0.1–0.7	×10^3^/µL	0.4	0.1
eos	0.2–1.6	×10^3^/µL	0	0
PLT	151–600	×10^3^/µL	52	5
Chemistry				
Total proteins	5.7–7.8	g/dL	7.6	6
Albumin	2.3–3.5	g/dL	3	1.8
ALT	22–84	IU/L	74	149
ALP	0–58	IU/L	45	15
TBil	<0.4	mg/dL	NT	3.6
GLU	71–148	mg/dL	140	37
TCho	95–259	mg/dL	154	193
BUN	17.6–32.8	mg/dL	22	78.8
Cre	0.9–2.1	mg/dL	1.4	2.6
Phosphorous	2.6–6	mg/dL	3.5	7.7
Calcium	8.8–11.9	mg/dL	10.3	9.1
Sodium	147–156	mmol/L	158	148
Potassium	3.4–4.6	mmol/L	4.7	3.4
Chloride	107–120	mmol/L	118	104

RBC, red blood cell; Hct, hematocrit; HGB, hemoglobin; MCV, mean corpuscular hemoglobin; MCHC, mean corpuscular hemoglobin concentration; WBC, white blood cell; band-N, stab cell; seg-N, segmented cell; lym, lymphocyte; mon, monocyte; eos, eosinophil; PLT, platelet count; ALT, alanine transaminase; ALP, alkaline phosphatase; TBil, total bilirubin; GLU, glucose; TCho, total cholesterol; BUN, blood urea nitrogen; Cre, creatinine; NT, not tested.

**Table 2:** Drug susceptibility of the LMN08 strain isolated in this study.

Antibiotic	MIC	Interpretation
Minocycline	4	S
Ceftazidime	8	S
Levofloxacin	≤0.5	S
Trimethoprim–sulfamethoxazole	≤2/38	S
Meropenem	≤1	S
Piperacillin	>64	N/A
Gentamicin	>8	N/A
Tobramycin	>8	N/A
Amikacin	>32	N/A
Cefozopran	>16	N/A
Cefepime	>16	N/A
Ciprofloxacin	0.5	N/A
Fosfomycin	>16	N/A
Azidothymidine	>16	N/A
Tazobactam–piperacillin	≤8	N/A
Imipenem	4	N/A

MIC, minimum inhibitory concentration; S, susceptible; N/A, not available.

## Data Availability

The nucleotide sequence obtained in this study was submitted to GenBank (accession number LC834785).
